# Glioblastoma Tumor Microenvironment: An Important Modulator for Tumoral Progression and Therapy Resistance

**DOI:** 10.3390/cimb46090588

**Published:** 2024-09-05

**Authors:** Ligia Gabriela Tataranu, Serban Turliuc, Amira Kamel, Radu Eugen Rizea, Anica Dricu, Georgiana-Adeline Staicu, Stefania Carina Baloi, Silvia Mara Baez Rodriguez, Andrada Ioana Maria Manole

**Affiliations:** 1 Neurosurgical Department, University of Medicine and Pharmacy “Carol Davila”, 020022 Bucharest, Romania; rizea.radu.eugen@gmail.com; 2 Neurosurgical Department, Clinical Emergency Hospital “Bagdasar-Arseni”, 041915 Bucharest, Romania; kamel.amyra@yahoo.com (A.K.); mara.silvia@icloud.com (S.M.B.R.); oana.andrada92@gmail.com (A.I.M.M.); 3 Medical Department, University of Medicine and Pharmacy “G. T. Popa”, 700115 Iasi, Romania; serban_turliuc@yahoo.com; 4 Biochemistry Department, University of Medicine and Pharmacy, 200349 Craiova, Romania; anica.dricu@live.co.uk (A.D.); adstaicu@gmail.com (G.-A.S.); carina_baloi@yahoo.com (S.C.B.)

**Keywords:** glioblastoma, tumor microenvironment, molecular diagnostic, extracellular matrix

## Abstract

The race to find an effective treatment for glioblastoma (GBM) remains a critical topic, because of its high aggressivity and impact on survival and the quality of life. Currently, due to GBM’s high heterogeneity, the conventional treatment success rate and response to therapy are relatively low, with a median survival rate of less than 20 months. A new point of view can be provided by the comprehension of the tumor microenvironment (TME) in pursuance of the development of new therapeutic strategies to aim for a longer survival rate with an improved quality of life and longer disease-free interval (DFI). The main components of the GBM TME are represented by the extracellular matrix (ECM), glioma cells and glioma stem cells (GSCs), immune cells (microglia, macrophages, neutrophils, lymphocytes), neuronal cells, all of them having dynamic interactions and being able to influence the tumoral growth, progression, and drug resistance thus being a potential therapeutic target. This paper will review the latest research on the GBM TME and the potential therapeutic targets to form an up-to-date strategy.

## 1. Introduction

The fifth edition of the World Health Organization Classification of Tumors of the Central Nervous System (CNS) published in 2021 introduced a few major changes, upgrading the importance of molecular diagnostics in tumor classification [1] and transforming prognostic biomarkers in tumor-grade parameters [2]. GBM is the most prevalent and aggressive malignant primary brain tumor, as glioma stem cells (GSCs) have the ability to develop into tumors with a high grade of heterogenicity and a high potential for self-renewal, factors that contribute to progression, aggressiveness, and therapeutic resistance [3].

Currently, conventional GBM therapy focuses on achieving a surgical maximal resection followed by radiation and chemotherapy with temozolomide (TMZ), according to the Stupp protocol [4], in order to maintain a better quality of life and prolong the survival rates. Even with the latest achievements and intense research, curative treatment is still not available and GBM median survival rates remain low, at approximately 14–20 months with near universal lethality [5].

Establishing the structure of the GBM microenvironment is an important target point in any effective therapeutic strategy, given the demonstrated differences between even same-grade tumors [6] and the need to determine unique cellular characteristics in each patient. The TME is a dynamic structure that collects normal cells, cancer cells, secreted factors, and an extracellular matrix that coordinates tumoral growth, invasion, and angiogenesis [7]. Each tumor has a unique immune component with an important role in tumoral progression, thus immunotherapeutic strategies to obtain disease control are being investigated [8].

Hypoxia and the disruption of the normal mitochondrial function are responsible for an acidotic environment and the presence of acidic waste products such as glutamic acid, glutamic acid, and lactic acid generated by the tumoral cells are responsible for resistance, invasion, and progression [9]. Also, an important role in drug resistance, progression, and aggressiveness is attributed to the disruption of the TME during the initial phases of the treatment, namely surgical resection, chemotherapy, and radiotherapy [10].

To determine the most efficient therapeutic strategy, we must understand not only the components of the GBM TME but also how they interact, their role in therapy resistance, and how we can interfere in order to stop tumoral progression.

## 2. Glioblastoma Microenvironment—Main Features

In recent years, our information about tumorigenesis has evolved and we know that genetics alone is not able to generate and maintain tumoral growth, a fundamental role being attributed to the TME, with GBM not being an exception [11].

GBM TME has been, for many years, a subject of debate in the journey of finding an effective therapeutic pathway. It is important to understand that the interaction between the TME and tumoral cells greatly influences the invasiveness, tumoral growth, and molecular heterogenicity of GBM [6].

**Figure 1:**
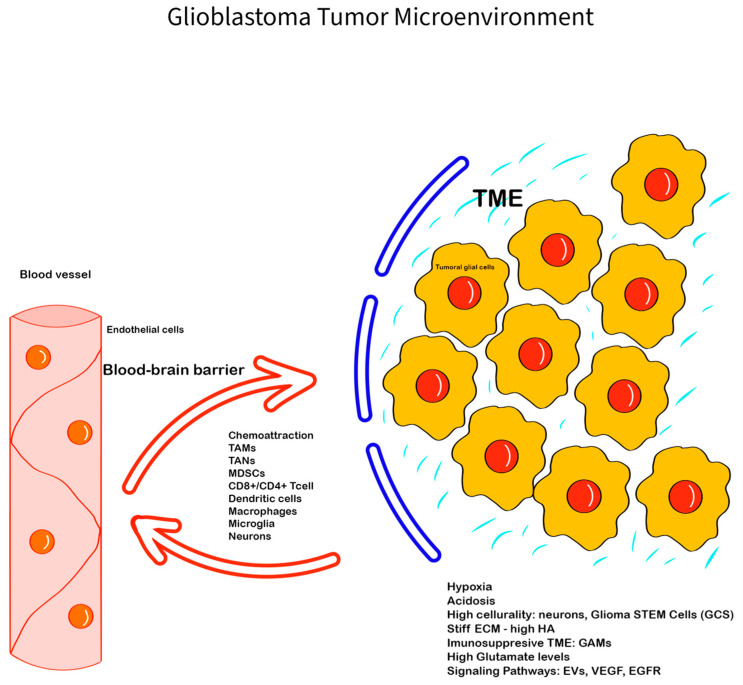
Glioblastoma Tumor Microenvironment: The GBM TME is complex, with a series of unique features, interactions, and components—the ECM, with glycoproteins, proteoglycans, and glycosaminoglycans (e.g., HA), has an important role in creating a barrier between the GBM TME and the normal tissue, promoting invasiveness; the immune cells (GAMs, TANs, monocytes, tumor-infiltrating lymphocytes), neuronal cells, glial cells, and glioma stem cells; chemokines, hormones, enzymes, EVs, and cell-communicating factors like VEGF, EGFR, and mediators like glutamate, with all of them being involved in creating an hypoxic and immunosuppressive TME that promotes tumoral progression and therapy resistance. The blood–brain barrier is also important in modulating the GBM TME components and can limit tissue drug availability, playing an important role in therapy resistance.

The main components of the GBM TME are the glioma cells and GSCs, immune cells, neuronal cells, the perivascular niche, communication factors, the extracellular matrix, and chemical components such as the oxygen levels and the pH [12] (Figure 1).

**Figure 2:**
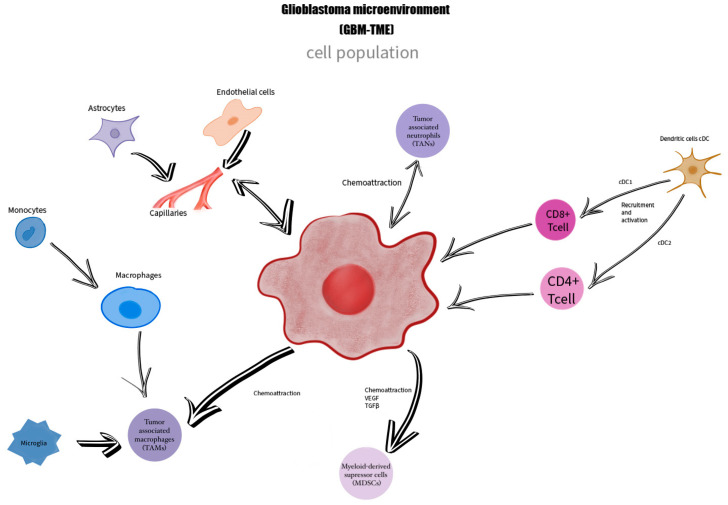
The GBM TME is formed by a heterogeneous cell population represented by glioblastoma cells, macrophages, monocytes, microglia—TAMs, tumor-associated neutrophiles—TANs, dendritic cells, astrocytes, endothelial cells, myeloid-derived suppressor cells MDSCs, who are in a close connection, being linked together by mediators and chemoattraction processes.

In the GBM TME, different types of neoplastic and non-neoplastic cells are involved, such as microglial cells, astrocytes, oligodendrocytes, leukocytes, macrophages, neuronal precursor cells, vascular cells, fibroblasts, dendritic cells, and endothelial cells, all of which have a role in tumor neogenesis through specific pathways [6]. The non-neoplastic cells form approximately 30% of the tumoral volume, and understanding the role of each one in the TME can contribute to the development of a new therapeutic target [13]. Astrocytes have different roles such as maintaining the brain–blood barrier’s integrity and supporting tissue repair, but they can also be transformed into tumor-associated reactive astrocytes (TARAs), promoting tumoral growth and invasiveness [10]. The tumor-associated macrophages (TAMs) are involved in tumoral modulation by producing different types of cytokines, growth factors (e.g., vascular endothelial growth factor), and other molecules [14,15] (Figure 2).

In addition, an important part of GBM TME is represented by the following extracellular components: extracellular matrix (ECM), soluble factors such as cytokines, chemokines, hormones, matrix remodeling enzymes, and growth factors involved in the cell-communicating mechanism, with all of them being involved in the specific characteristics and thus being potential therapeutic targets [12]. Current studies mention the presence and importance of extracellular vesicles (EVs) in the GBM TME and their potential involvement in increasing cell communication and the modulation of GBM aggressiveness [16].

The TME components and their interactions are current hotspots for therapeutic approaches, with the dynamic interaction between the abnormal tumoral cells, non-neoplastic cells, the ECM, and the immune system being essential for cancer progression [15,17].

We will further revise the main components of the GBM TME and their importance in tumoral proliferation, invasiveness, and therapy resistance, as well as the current and potential targeted therapies.

### 2.1. The Extracellular Matrix of GBM TME

Approximately 10–20% of brain volume is represented by the ECM, a condensed structure made of glycoproteins (e.g., fibronectin, tenascins TN-C, TN-R, TN-W, TN-X), glycosaminoglycans (e.g., hyaluronic acid—HA), proteoglycans (lectican family—aggrecan, brevican), and collagen IV, all with unique features [18,19]. The GBM ECM was initially considered to have a purely structural role. In recent years, it has been determined to have important tasks regarding the functional responses and cell communication in TME and is an active component [20]. The GBM ECM has a series of distinct characteristics such as an overexpression of its components; the lack of aggrecan; the presence of oncofetal proteins; and elevated levels of metalloproteinases (MMPs—matrix metalloproteinases) that increase density and promote invasion and angiogenesis, allowing the tumor to become stiffer and potentially able to diminish the diffusion of therapeutic drugs and neuroactive molecules [19].

In the dynamics of the TME, a key role is represented by the disruption and destruction of the normal ECM to promote cell migration and invasiveness. Also, the ECM can play a role in therapy resistance and local immunosuppression, with high levels of collagen being associated with immunotherapy resistance in different types of cancer [21]. Because of the unique immune-privileged features of the GBM TME, immune checkpoint inhibitors (CIs),which has managed to provide a new perspective in cancer therapy, failed to show significant clinical improvement, with the architecture of the ECM, especially the collagen, being linked with anti-programmed cell death-1 (PD1)/programmed cell death ligand-1 (PD-L1) resistance [21,22].

In the early stages of tumor growth, proteolytic enzymes such as MMPs, that destroy the local barrier, are overexpressed [23]. Researchers have intensively studied the role of MMPs in tumoral development and the GBM TME. MMP-2 and MMP-9 are associated with poor prognosis and overexpressed in the GBM [23,24]. MMP inhibitors such as marimastat were used in clinical trials in association with temozolamide, especially for recurrent and progressive GBMs, and had minimal favorable effects [25,26].

Tissue inhibitors of metalloproteinase (TIMP) are a family of proteins produced by different types of cells, including astrocytes, with a role in ECM breakdown, reshaping, and cell communication thus being associated with various types of tumors including the GBM [27,28]. These proteins are also a key component of the TME, being involved in synaptic plasticity and having pro-apoptotic and anti-angiogenetic roles as well [29]. The TIMP family is represented by four members, TIMP 1–4, with TIMP1 being upregulated in tumorigenesis, particularly in the GBM [30].

Proteoglycans are extracellular macromolecules involved in a series of tumoral processes like adhesion, migration, inflammation, and angiogenesis [20]. Researchers observed the upregulation of a series of molecules, including chondroitin sulfate proteoglycan (CSPG), the associated sulfated glycosaminoglycans (GAGs), and the related enzymes, in the tumor microenvironment (TME) [31]. The CSPG proteins such as Versican, one of the most abundant in the GBM ECM, have a role in cell migration and adhesion [20].

Tenascins (TN-C, TN-R, TN-W, and TN-X), with the main representative TN-C, are glycoproteins involved in cell migration, proliferation, and angiogenesis. They tend to be overexpressed in the GBM. Moreover, the level of TN-C can be related to tumoral proliferation, with studies mentioning a proportional relation between the TN-C levels and the aggressivity of the GBM [18,20]. Other glycoproteins that are found in large amounts in the GBM TME are fibulin-3 and fibronectin. Fibulin-3 helps cells stick together, grow, and resist chemotherapy by stopping p53-mediated apoptosis [20].

HA has a significant role in the GBM ECM, as the overexpression of HA and collagen is involved in the development of a limiting barrier for drugs and is associated with therapy resistance. In addition, Kiyokawa et al. associated a lower T cell infiltration in tumors with high amounts of HA and raised the possibility of using oncolytic virotherapy like ICOVIR17-mediated degradation of HA, in combination with Pembrolizumab, an antibody that blocks PD-1 protein on the T-cells membrane to increase the permeability of the therapeutic agent by altering the HA barrier [32].

GBM EMC can be a target for therapeutic strategies to prolong survival rates and improve quality of life in the fight against this aggressive type of cancer.

### 2.2. The Immune Component of GBM TME

The immune system regarding the CNS plays an important role in shaping the structure of GBM TME, being associated with progression and prognosis [27,33].

For a long time, the CNS was considered a privileged organ because of the blood–brain barrier (BBB) and its highly controlled inflammatory and immune responses [8]. This status can determine multiple limitations in treating GBM, as the BBB can restrict immune infiltration and create a profound immune-suppressive TME, thereby posing a challenge for newly developed immunotherapy. In recent years, immunotherapy had a positive impact on the survival time and life quality of patients with different types of cancer but has not been able to achieve the same effect on the GBM, mainly because of the immunosuppressive TME, heterogenicity, and low immunogenicity [34,35,36].

It can be speculated that the glymphatic system, a clearance system formed by glial cells located in the walls of dural sinuses [37], can be involved in the particular immune microenvironment of GBM despite not being directly connected with the brain parenchyma, but further studies need to be performed regarding its role in the modulation of the GBM TME [12,38].

Microglia, glioma-associated macrophages (GAMs), neutrophils, monocytes, and tumor-infiltrating lymphocytes represent approximately 50% of the tumor cellularity in the glioma immune microenvironment, coexisting with cancer cells, neurons, and glial cells [12].

Immune cells from the TME express cellular receptors to influence tumor cell phenotypes and cellular recruitment, shaping dynamic crosstalk that critically influences the TME [39]. The co-stimulatory and co-inhibitory molecules involved in T-cell regulation represent immune checkpoints, and tumoral cells use them to suppress and escape an immune attack [40,41].

Microglia and glioma-associated macrophages (GAMs) are the predominant populations of immune cells in the GBM TME, playing a crucial role in local immunity with prompt response in the presence of antigens or lesions [40,42]. In the GBM, because of local inflammation, the activation of the microglial cells is performed, supported by the bone marrow-derived macrophages (BMDMs), forming approximately one-third of the tumor mass with pro-tumorigenic and immunosuppressive actions in the GBM TME [43,44]. Furthermore, tumor necrosis factor-alpha (TNF-α) is expressed by the microglial cells, facilitating immune infiltration from the periphery [43]. GAMs are recruited by glioma-cell-derived factors like CCL2, CXCL1, SFD-1, CSF-1, CM-CSF, GDNF, and EGF [43]. Moreover, GAMs are able to produce different types of substances like cytokines with anti-inflammatory potential (IL4, IL10, Transforming growth factor-beta—TGFβ), angiogenesis factors (Vascular Endothelial Growth Factor—VEGF, IL8), pro-tumorigenic factors (Insulin-like Growth Factor 1—IGF-1, epidermal growth factor—EGF, platelet-derived growth factor—PDGF), with all of them having important and specific roles in the modulation of GBM TME and in therapy resistance; therefore, they are a potentially therapeutic target, especially since the presence of GAMs was associated with a poor prognosis [12,43]. In short, tumor-associated macrophages (TAMs) consist of up to 30% of the GBM TME and tend to stimulate tumor proliferation. Moreover, they produce low pro-inflammatory cytokine levels and can suppress cluster differentiation 8 (CD8) + T cell activity [45].

Myeloid-derived suppressive cells (MDSCs), a type of bone marrow-derived cells found in the blood and lymphatic organs of patients with a GBM were associated with an immunosuppressive effect by altering the normal function of T cells like the natural killer T cells (NK) or cytotoxic T lymphocytes (CTLs), depleting essential amino acids, such as L-Arginine, or boosting the production of the reactive oxygen species (ROS) [46,47].

Neutrophiles, the most abundant type of leukocyte in the bloodstream are also a part of the immune microenvironment of GBM. For instance, Gabrusiewicz et al. observed a rise in the CD11b+CD16+ neutrophils in the peripheral blood of GBM patients with immunosuppressive function [48]. With their elastase and metalloprotease secretion, they support tumoral progression in GBM. In addition, an elevated neutrophiles-to-lymphocytes ratio has been associated with poor clinical outcomes in the GBM [49].

Natural killer (NK) cells in the GBM are one of the least represented types of immune cell populations, as the composition of the GBM TME plays an important role in suppressing their function [12]. NK cells have a cytolytic activity that can contribute to efficient immune surveillance, being the central point of the development of immune strategies due to their natural cytotoxicity, deep penetration, and resistance to immune suppression [34,50]. In an isocitrate dehydrogenase (IDH) mutant GBM, the NK surveillance is altered, having a significant role in increasing aggressivity [51]. NK remains a standing point for the development of new potentially effective immune strategies like chimeric antigen receptors (CARs) [50].

### 2.3. Neuronal and Glial Component of GBM-TME

The CNS is comprised of neurons and glial cells with complex and specific interactions and are an important part of the GBM TME. Intense research has been performed to determine the exact mechanisms of communication between the GBM cells and the normal cells of the CNS by synapses, paracrine stimulation, mediators, and their role in progression and invasiveness [12].

We already know that the GBM can integrate into neuronal pathways, and some of the mediated synapses may play a role in tumor progression and growth through paracrine stimulation, making them potential targets for GBM therapy [52]. Researchers have studied the brain-derived neurotrophic factor (BDNF/abrineurin/neurotrophin), which plays a role in differentiation and apoptosis by the overexpression of transmembrane tyrosine kinase B (TrkB) in gliomas [53,54], and NLGN3 (neuroligin 3), a cell-adhesion molecule [12], but further research is necessary to unveil possible therapeutic strategies.

Glutamate is another intensely studied neurotransmitter of the GBM TME involved in neuroexcitability, excitotoxic neuronal cell death, seizures, and tumoral progression. For more than 25 years, it has been established that GBM cells secrete high amounts of glutamate, as research has demonstrated an increase in the extracellular concentration in glutamate-depleted culture medium from 1 µM to approximately 500 µM in less than 48 h [55]. In vivo studies also revealed the increased levels of glutamate produced by the GBM cells. Marcus et al. demonstrated using microdialysis when the level of glutamate is 30 times higher in glioma tissue compared with peritumoral tissue [56]. The same study showed that the glioma resection margin had a significantly higher IL-8 concentration, as well as a higher MMP-2/TIMP-1 ratio compared to peritumoral tissue. This, being metabolically extremely active, favors invasion and angiogenesis at the tumor margin, thereby promoting invasiveness by disrupting the barrier between the TME and normal brain tissue [56].

Glutamate is thought to be a major factor in the malignant behavior of GBM, contributing to the microenvironment’s toxicity [57]. This neuromediator stimulates the surrounding neurons by activating the N-methyl-D-aspartate (NMDA) and non-NMDA receptors that allow for an Na+ influx, leading to depolarization and cell-to-cell communication [58]. The GBM cells also express α-amino-3-hydroxy-5-methyl-4-isoxazolepropionate (AMPA) receptors that are involved in Ca2+ permeability [57]. The Ca2+ influx through cell receptors can result in cell death or activation of metabolic cascades [58].

Van Vuurden et al. speculated that the downregulation and modified function of AMPA-type glutamate receptors are responsible for the survival of GBM cells in a high-glutamate environment [59] and the manipulation of AMPA receptors can alter glioma growth, invasion, and toxicity [57].

### 2.4. Communication Factors

The GBM cells need to modulate their metabolic activity in order to maintain their growth and development. Communication factors are involved in pathogenesis, with each of them having a significant role in the development of new drugs and therapeutic approaches.

EVs are cell-derived structures surrounded by membranes that transport molecules and deliver them to recipient cells, making them key components of TME cell communication [60].

EVs carry encapsulated simple materials such as proteins, lipids, and more complex ones like nucleic acids or histones [60]. Recently, the EV classification has undergone changes. Classically, they were described as microvesicles (40–1000 nm), exosomes (50–200 nm), apoptotic bodies (50–2000 nm), and oncosomes (>1 µm) [60,61]. Recently, matrix, autophagic, and stress EVs were described to enlighten the specific pathways and the role of EVs in the TME and cancer immunomodulation [60]. It has been established that EVs are involved in dynamic communication by transferring oncogenic proteins and mRNA between cancer cells and tumor-associated cells (TACs) such as microglia, T cells, and astrocytes, mediating cell proliferation, angiogenesis, migration, and invasion. These structures are also involved in the immunomodulation and resistance to treatments such as chemoradiotherapy [61]. Furthermore, apoptotic EVs (apoEVs), which are enriched with spliceosomal proteins and non-coding RNA, can induce proliferation and therapy resistance by preparing neighboring tumoral cells for aggressive factors [62]. One of the most abundant proteins in the GBM EVs is Annexin A2 [63], a protein involved in angiogenesis and tumoral progression [64]. Understanding the EVs’ role in cell communication and the TME may reveal a future target for GBM treatment.

### 2.5. The Perivascular Niche (PVN)

A key component of the GBM TME is represented by the dimensional relationship between the GBM cells and the endothelial cells that form the PVN [65]. An important feature of the GBM is represented by angiogenesis, which plays a role in recurrence, proliferation, and invasion. The PVN of the TME is characterized by disrupted and fragile microvessels [65,66,67].

Growth factors have a critical role in the GBM TME, and the dysfunction of growth factors is associated with GBM progression and therapy resistance [68].

The vascular endothelial growth factor (VEGF) is part of a family of proteins, the superfamily of platelet-derived growth factors. VEGF is involved in tumor angiogenesis, being a modulator of endothelial cell growth, a permeabilizing agent, and an MMP activator [69,70]. VEGF is a factor for tumoral progression and the development of blood vessels in the GBM, and its concentration can influence microglia and macrophages to assist in angiogenesis [71]. In the GBM TME, the newly formed vessels are fragile and predisposed to rupture, interfering with the functional BBB, affecting the vascular permeability, causing edema, altering the immune response, and disrupting the barrier between the tumoral and normal brain tissue, favoring invasiveness [72].

A theoretically efficient therapeutic target can be pointed to the PVN, impacting pathological vascular proliferation and using angiogenesis inhibitors to slow the tumoral progression [72]. Currently, targeting angiogenesis with Bevacizumab (Bev), an anti-VEGF monoclonal antibody, to limit the growth of the GBM is a reliable therapeutic option, even though it failed to extend survival rates, despite influencing the extension of progression-free survival in some studies [73]. Bev treatment impacts the TME by affecting tumor angiogenesis and therefore the oxygen and nutrient supply, consequently shaping the tumor’s glycolytic metabolism [74]. The development of a hypoxic TME as well as the metabolic remodeling would lead to an increase in lactate and tumoral cell invasion into the normal tissue and thus confer resistance to Bev treatment [74,75].

Another growth factor is represented by the epidermal growth factor receptor (EGFR), an oncogenic tyrosine-kinase receptor formed by an extracellular domain (ECD), a transmembrane domain (TMD), an intracellular juxta membrane domain (JMD), a tyrosine kinase, and a C-terminal end that was one of the first oncogenes identified in the GBM [76]. EGFR is present in more than 50% of the GBM, along with other active mutated variants like variant II (EGFRvII), III (EGFRvIII), and IV (EGFRvIV), involved in tumoral progression, angiogenesis, and treatment resistance [77]. EGFR plays a significant role in GBM progression by influencing glutamine metabolism and regulating the MYC proto-oncogene [76]. EGFRvIII is present in almost one-third of the GBM and can be used as a GBM-specific marker [76]. Oncogenic EGFR and EGFRvIII can be carried by EVs [61].

A set of anti-EGFR clinical trials were performed and produced mixed results. For instance, Nitozumab, an antibody targeting the L2 domain of EGFR, presented some responses in the treatment of pediatric diffuse intrinsic pontine glioma [78].

### 2.6. Hypoxia and Hypoxia-Inducible Factors (HIFs)

According to the Pasteur effect, normal cells suppress glycolysis in the presence of oxygen, decreasing lactate accumulation in cells. On the other hand, cancer cells have an altered metabolism in which they prefer glycolysis and the fermentation of glucose to lactate in the presence of oxygen, despite the normal functioning mitochondria, known as the Warburg effect [79]. The high amount of lactate produced in the tumoral cells, according to the Warburg effect, accumulates in the tissue, lowering the pH (acidotic environment), inhibiting the antitumor immune responses, and thus becoming a metabolic hallmark of the TME that favors tumoral growth and progression [80].

One of the main characteristics of the GBM TME is hypoxia, determined by an inadequate blood supply [72]. VEGF is known for inducing hyper-proliferation of the endothelial cells, resulting in abnormal blood vessels that can be easily disrupted [69], leading to a hypoxic microenvironment that promotes the invasion of tumoral cells. The hypoxic TME is also a challenge for drug delivery and immune cell distribution inside the tumor.

## 3. Targeted Therapies in GBM TME

Despite the technological advances, intensive studies, and detailed genetic characterization of the GBM, the current prognosis remains low, with a 5% survival rate at 5 years [1,81]. Different approaches were used to find an effective treatment and improve the survival rate, as in other types of cancer. Because TME regulates the GBM progression, affecting treatment response, drug resistance, and survival, therapies targeting and revitalizing the TME can be the key to increasing overall survival and maintaining a good quality of life [33,82]. Immunotherapeutic strategies are now under investigation and can transform the future of the GBM therapeutic approach, even if most attempts have not managed to obtain significant success for now, mostly because of the GBM’s immunosuppressive TME and its propriety to develop resistance [83]. Selective targeting of the GBM TME can be a reliable option, and further studies are necessary.

Targeted therapies for pathways affected by the GBM’s common mutations, such as TP53, IDH1, NF1, and EGFR, did not return with a favorable outcome [81]. For example, Rindopepimut, also known as CDX-110, a vaccine that uses a proteinaceous immunogen against EGFRvIII, showed promising results in preclinical studies but did not manage to pass a phase III clinical trial because it was not able to demonstrate its capability to increase survival [84,85,86]. Anti-VEGF drugs such as bevacizumab, cediranib, and enzastaurin were used in phase III clinical trials but did not show superior efficacy [87,88,89].

Marimastat, an MMP inhibitor, entered a phase II trial in association with temozolomide in 2002 for recurrent and progressive glioblastoma multiforme but did not manage to demonstrate further clinical impact [25].

Targeting the RNA splicing events that produce apoEVs was also studied to diminish the aggressive changes that occur in the GBM TME because approximately 70% of the cell population of GBM is represented by apoptotic cells [51]. In vitro studies about apoptosis regulator Bcl-extra (BCLX) pre-mRNA splicing modulation were able to produce promising results in inhibiting the GBM, alone or in combination with radiotherapy [90].

Tumor-treating fields (TTFields) that interfere with the division rate of tumoral cells are currently under extensive investigation, with some entering phase II clinical trials [91,92].

ICIs are currently under investigation for GBM treatment. Compared to chemotherapy, which directly breaks down tumoral cells, ICIs enhance the immune response and can be useful in the immunosuppressive GBM TME to achieve a successful therapeutic target [39]. Nivolumab, a PD-1 ICI, was tested in combination with radiotherapy, but the phase III studies were not able to show significant changes in overall survival [93]. Ipilimumab, an anti-cytotoxic T-lymphocyte-associated antigen 4 (CTLA4) antibody, is also currently being tested for GBM [94].

CAR-T therapies, another type of immunotherapy made by reprograming T cells to express chimeric antigen receptors (CARs) in order to bind to the antigens present on the cancer cells are another potential target therapy in GBM, having better BBB penetration and being able to directly destroy glioma cells without the help of the already suppressed immune system, mutant EGFR, interleukin-13 receptor α chain variant 2 (IL13Rα2), and human epidermal growth factor receptor 2 (HER2), which are the most targeted antigens [95]. Considering the fact that the GBM comprise solid tumors and, in the GBM TME, macrophages and neutrophils are one of the main immune populations, they can be a useful tool for the development of new therapeutic targets. For example, the possibility of using macrophages from human pluripotent stem cells (hPSCs) to generate CAR hPSC-derived macrophages was used in in vitro studies with promising results [96]. Also, Jin et al. suggested that one of the keys to stopping the progression and control of this aggressive type of cancer may be in the transformation of the immune cells population of the GBM TME from pro-tumorigenic to anti-tumorigenic, especially the macrophages [96]. An important factor in GBM recurrence is represented by the GCS and using a cavity-injectable nanoporter–hydrogel superstructure in order to prevent regrowth shows promising results in orthotopic mouse models [97].

### Active Clinical Trials

The GBM TME represents a hot topic for the development of new therapeutic strategies, with clinical trials exploring the microenvironment currently in progress (Table 1).

## 4. Conclusions

The GBM TME is a complex structure with unique factors, interactions, and heterogeneity that must be individualized to pursue an efficient therapeutic path. Given its aggressiveness and lack of effective therapeutic strategies, for now, the GBM remains a challenge despite significant research on the molecular basis and cell interactions between immune cells, neurons, normal glial cells, GSCs, and glioma cancer cells that are part of the GBM TME. To obtain preclinical targets and translate them into an effective therapeutic strategy in the future, we must understand the TME and the cell interactions that are involved in the modulation of tumoral growth and progression.

## Figures and Tables

**Table 1:** International active clinical trials investigating GBM and its microenvironment.

Country	Title	Year
United States of America (North America)	A Phase 1b/2a Study of ACT001 and Anti-PD-1 in Patients with Surgically Accessible Recurrent Glioblastoma Multiforme	Started on 22 September 2021
Germany (Europe)	Characterization of Metabolic Changes in the Glioma Tumor Tissue Induced by Transient Fasting (ERGO3)	Started on 19 August 2020
China (Asia)	The Study of Microglia/Macrophages Involved Dynamic Evolution of Glioma Microenvironment and the Function and Visualization of Targeted Molecules of Glioma	Started on 9 November 2020
United States of America (North America)	AB154 Combined With AB122 for Recurrent Glioblastoma	Started on 21 April 2021
United States of America (North America)	Surgical Nivolumab And Ipilimumab For Recurrent GBM	Started on 1 February 2021
China (Asia)	Testing the Addition of the Immune Therapy Drugs, Tocilizumab and Atezolizumab, to Radiation Therapy for Recurrent Glioblastoma	Started on 11 March 2022
United Kingdom (Europe)	A Trial of Ipatasertib in Combination With Atezolizumab (IceCAP)	Started on 13 August 2018

## Data Availability

No new data were created or analyzed in this study. Data sharing is not applicable to this article.
